# Hepatitis E Virus ORF2 Inhibits RIG-I Mediated Interferon Response

**DOI:** 10.3389/fmicb.2020.00656

**Published:** 2020-04-15

**Authors:** Smita Hingane, Nishant Joshi, Milan Surjit, C. T. Ranjith-Kumar

**Affiliations:** ^1^Virology Laboratory, Vaccine and Infectious Disease Research Centre, Translational Health Science and Technology Institute, Gurgaon, India; ^2^University School of Biotechnology, Guru Gobind Singh Indraprastha University, Dwarka, India

**Keywords:** HEV, ORF2, RIG-I, interferon, innate immunity

## Abstract

Understanding the dynamics of host innate immune responses against a pathogen marks the first step toward developing intervention strategies against the pathogen. The cytosolic pattern recognition receptor retinoic acid-inducible gene I (RIG-I) has been shown to be the major innate immune sensor for hepatitis E virus (HEV). Here, we show that HEV capsid protein (ORF2), a 660 amino acid long protein, interferes with the RIG-I signaling. Interestingly, only the full length ORF2 protein but not the 112-608 ORF2 protein inhibited RIG-I dependent interferon response. Both synthetic agonist and virus induced RIG-I activation was modulated by ORF2. Interference of interferon response was confirmed by reporter assays involving different interferon inducible promoters, qRT PCR, ELISA, and immunofluorescence microscopy. Neither glycosylation nor dimerization of the ORF2 protein had any effect on the observed inhibition. Further analyses revealed that the ORF2 protein antagonized Toll-like receptor (TLR) pathways as well. ORF2 inhibited signaling by RIG-I and TLR adapters, IPS-1, MyD88, and TRIF but was unable to inhibit activation by ectopically expressed IRF3 suggesting that it may be acting at a site upstream of IRF3 and downstream of adapter proteins. Our data uncover a new mechanism by which HEV may interfere with the host antiviral signaling.

## Introduction

Hepatitis E virus (HEV) has emerged as a leading cause of viral hepatitis since its discovery in the 1980s (Purcell and Emerson, 2008). The disease is self-limiting in healthy individuals but leads to chronicity and severe liver damage in patients with compromised immune system such as organ transplant patients (Melgaço et al., 2018). Due to its small size, HEV can easily cross the blood–brain barrier causing neuropathological manifestations (Shi et al., 2016). In rare cases, other extrahepatic manifestations such as musculoskeletal, hematological, renal, and immunological diseases have also been reported (Bazerbachi et al., 2015). A recent report estimated that 70,000 HEV-related deaths occur each year (Melgaço et al., 2018). The case fatality rate is particularly high in pregnant women (25–30%) (Melgaço et al., 2018).

Current anti-HEV treatments are limited to ribavirin therapy which cannot be administered to pregnant women due to its teratogenic effects (Todt et al., 2018). Additionally, in chronic cases it leads to ribavirin induced anemia in 50% of the patients (Kamar et al., 2016; Van De Garde et al., 2017). PEGylated interferon is used in combination or as an alternative to ribavirin. However, it cannot be used for the high risk group of transplant patients due to fears of graft rejection (Kamar et al., 2016). Recent years have seen an increase in the number of reported HEV cases due to improved detection techniques, highlighting the need for developing better interventions against HEV.

Understanding viral interactions with the host and their roles in immune-modulation events during infection could lead to improved treatment regimes. The cytosolic pattern recognition receptor retinoic acid-inducible gene I (RIG-I) has been shown to be the major innate immune sensor for HEV (Xu et al., 2017). Classically, activation of RIG-I upon recognition of the viral RNA leads to its association to its adapter protein IPS-1 (also known as MAVS, VISA, and Cardif) and induction of a downstream cytokine, interferon beta (IFN-β) through a signaling cascade. Although the HEV RNA is recognized by RIG-I, downstream production of IFN-β upon recognition was low during HEV infection (Yin et al., 2017). These observations indicate that HEV might be modulating the RIG-I pathway to delay or subdue the host antiviral response. Furthermore, HEV ORF1 proteins, such as X, PCP (Nan et al., 2014b), and methyl transferase (MeT) (Bagdassarian et al., 2018; Kang et al., 2018) have been shown to lower IFN-β induction by inhibiting the RIG-I signaling. HEV ORF2 has been shown to inhibit the NF-κB signaling pathway by preventing proteasomal degradation of the IκBα protein (Surjit et al., 2012).

Here, we report that the full length HEV ORF2 protein (FL ORF2) inhibits RIG-I signaling in mammalian cells. Further characterization revealed that ORF2 interferes with the toll-like receptor mediated signaling as well. In the presence of FL ORF2, IFN-β induction and NF-κB activation are significantly lowered.

## Materials and Methods

### Cloning and Site Directed Mutagenesis

All the HEV plasmids were constructed by PCR amplification of HEV protein domains from genotype 1 HEV (HEV-1, pSK-HEV2) and genotype 3 HEV (HEV-3, pSK-P6). HEV replicons were a kind gift from Dr. Suzanne Emerson, National Institute of Allergy and Infectious Diseases, NIH, Maryland, United States. Digested amplicons were cloned into linearized pUNO-mcs vector (Invivogen) at specified sites with Flag, Myc, or HA epitope tags (PCP, RdRp, and ORF2 with the FLAG tag, MeT and helicase with the myc tag, and Domains X, Y and ORF3 with the HA tag). pUNO-IRF3 HA was subcloned from a commercially available vector. Clones were confirmed by restriction digestion and DNA sequencing. pUNO RIG-I, pUNO IPS-1, and pUNO MyD88 were purchased from Invivogen (United States). pcDNA-TRIF myc was a kind gift from Dr. Stanley Lemon, University of North Carolina, United States. IFN-β Firefly Luc reporter plasmid was a kind gift from Dr. R. Lin, McGill University, Canada. NF-κB Firefly Luc, ISRE Firefly Luc, ISG56 Firefly Luc, pRLTKLuc *Renilla* and cmvRLLuc *Renilla* reporter plasmids were purchased from Promega. Plasmid information is given in Supplementary Table S1. A detailed list of primers is given in Supplementary Table S2.

Point mutations were introduced at different positions as indicated using PCR-based site directed mutagenesis to generate the glycosylation and dimerization mutants. Details of all the primers used to generate mutants are given in Supplementary Table S3. Clones were sequenced to confirm successful mutagenesis.

### SDS PAGE and Western Blotting

For SDS PAGE, cell lysates in laemmli buffer (60 mM Tris-Cl Buffer pH 6.8, 2% SDS, 10% glycerol, 0.01% BPB, 0.1% β-mercaptoethanol) were incubated at 95°C for 3 min prior to loading and separated on 8–12% acrylamide gels with 0.1% SDS. Separated proteins were transferred to a PVDF membrane. Blocking was done using 5% non-fat milk for 1 h at room temperature. Primary antibody incubations were done for 16 h at 4°C in 5% blocking buffer containing the respective primary antibody at 1:1000 dilution. Proteins were detected using appropriate HRP tagged secondary antibodies (1:5000).

### Maintenance of Cell Lines and Transfections

HEK293T and Huh7 (WT and stable) cells were maintained in Dulbeco’s Modified Eagle Medium (DMEM) Glutamax supplemented with 10% FBS with penicillin and streptomycin at 37°C, 5% CO2. Stable cells were maintained in 4 μg/ml of blasticidin. DMEM was replaced with RPMI medium for THP-1 cells all other conditions were constant.

For transfections, cells were seeded at 70–80% confluency. Lipofectamine 2000 transfection reagent (Life Technologies) was used for DNA transfection at 1:1 ratio (1 μl Lipofectamine per μg of DNA). DNA concentrations are mentioned individually for each experiment in figure legends.

### Virus Infections

Purified Sendai virus (SeV) was a kind gift from Prof. Debi P Sarkar, University of Delhi, India. Cells were infected at an experimentally optimized dose of 40 HAU/ml for HEK293T and 100 HAU/ml for Huh7. All infections were done for 12–16 h in serum free DMEM containing penicillin and streptomycin.

Purified Japanese Encephalitis virus (JEV) was a kind gift from Prof. Sudhanshu Vrati, Regional Centre for Biotechnology, Faridabad, India. Cells were infected with JEV at 0.5 multiplicity of infection (MOI). Infections were done in serum free DMEM containing penicillin and streptomycin for 3–4 h and replaced with DMEM with serum containing penicillin and streptomycin. Cells were incubated for 12 h to ensure optimum induction.

### Luciferase Assays

Firefly luciferase cloned under the IFN-β promoter (IFN-β Luc) was used as the reporter for measuring IFN-β promoter induction. *Renilla* luciferase cloned under thymidine kinase promoter (pRLTKLuc) or CMV promoter (CMVRL Luc) was used as an internal control reporter for HEK293T or Huh7 cells, respectively. For RIG-I assay, RIG-I plasmid and reporter plasmids were transfected into HEK293T or Huh7 cells. 24 h post-transfection, a synthetic 5’ triphosphorylated small double-stranded RNA (3pdsR27), SeV, or JEV (as described for individual experiment) were used to induce the pathway. RIG-I was replaced with the IPS-1 plasmid for IPS-1 assay, myc-TRIF for TRIF assay, MyD88 for MyD88 assay, and HA-IRF3 for IRF3 assay. IPS-1 or IRF3 over-expression results in constitutive activation of IFN-β promoter. Wherever applicable, individual HEV clones were transfected along with the corresponding plasmids described above. Luciferase activity was measured 24 h post-transfection using Promega Dual Glo luciferase assay kit following manufacturer’s protocol. Firefly luciferase values were normalized with *Renilla* luciferase values and the data were plotted as % fold change where the test samples were compared to the induced positive control values taken as 100%.

### RNA Isolation and Quantitative RT (qRT) PCR

Total RNA was isolated from HEK293T or Huh7 cells using TRIzol (ThermoFisher Scientific, United States) reagent, as per manufacturer’s instructions. 1 μg of the RNA was used for cDNA synthesis with random hexamers using the GoScript first strand synthesis kit (Promega, United States). Gene specific primers were used to quantify IFN-β, ISG56, and NF-κB transcripts by SYBR green-based relative quantitation using the LC96 Real Time PCR machine (Roche, Life Science, United States). Test gene Ct values were normalized to the GAPDH Ct values and results were plotted using the ΔΔCt method. List of primers is present in Supplementary Table S4.

### Generation of Stable Cell Lines

Stable cell lines were created in human hepatoma cell line, Huh7. FLAG-tagged HEV-1 FL ORF2, 112-608 ORF2 constructs or empty vector were digested with *Not*I restriction enzyme. 2 μg of the purified linearized plasmids were transfected into Huh7 cells using lipofectamine 2000 reagent at 1:1 ratio (Life Technologies, United States). 24 h post-transfection, media was replaced with DMEM containing 10% FBS and 4 μg/ml blasticidin, repeating after every 72 h till cells in the un-transfected control were completely dead. The blasticidin resistant cells were propagated under continued blasticidin selection. Presence of ORF2 proteins was confirmed by western blot every 2 weeks. Cells were said to be stably expressing the proteins when it was consistently observed for more than 2 months.

### Immunofluorescence Assay

Huh7 cells transiently transfected with the FL ORF2 and 112-608 ORF2 plasmids were seeded on coverslips at 60% confluency. Cells were mounted in ProLong anti-fade gold reagent containing DAPI (ThermoFisher Scientific, United States). Cells were fixed with 2% paraformaldehyde in 1X PBS and ORF2 proteins were visualized with goat Anti-FLAG primary and anti-goat Alexa fluor 596 secondary antibodies.

For visualization of IRF3 translocation, pUNO Huh7, ORF2 Huh7, and 112-608 Huh7 stable cells were seeded on coverslips at 60% confluency and infected with JEV at 0.5 MOI. Cells were then fixed with 2% paraformaldehyde in 1X PBS. Rabbit anti-IRF3, mouse anti-JEV, and goat anti-FLAG antibodies were used as primary. Anti-rabbit Alexa-488 and an anti mouse Alexa-596 secondary antibodies were used. Cells were mounted in ProLong anti-fade gold reagent containing DAPI (ThermoFisher Scientific, United States). Slides were visualized using the Olympus FV3000 confocal microscope.

### ELISA

Undifferentiated THP-1 cells were transfected with the FL ORF2 or 112-608 ORF2 plasmid along with a vector control. SeV infection was given at 40 HAU/ml 12 h post-transfection. Media was collected from the samples 24 h post-infection by centrifugation at 1000 *g*, 20 min, 4°C. 100 μl of the media was used to quantify secreted IFN-β protein levels using a sandwich ELISA kit (SEA222Hu, Aspira chemical, United States) as per manufacturer’s instructions.

Purified IFN-β protein standard provided in the kit was processed in the same way as the test samples to obtain the standard curve (at concentrations mentioned in the plot given in Supplementary Figure S6A). Values of the test samples were plotted as absolute values of IFN-β protein (pg/ml) deduced from the standard curve.

### Statistical Analysis

Each experiment was performed at least thrice in triplicates. *P*-values were calculated using unpaired Student’s *t*-tests.

## Results

### IFN-β Production Is Inhibited by the HEV ORF2 Protein

**FIGURE 1:**
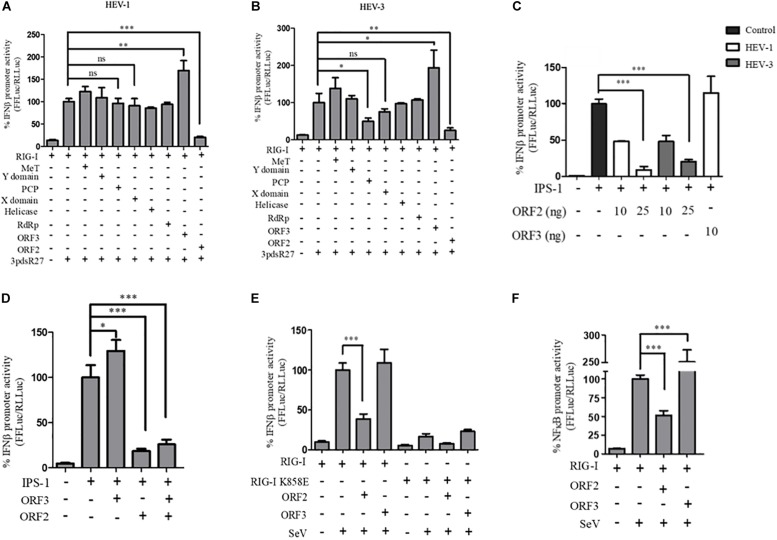
HEV ORF2 protein inhibits IFN-β promoter activation by downregulating the RIG-I pathway. **(A)** Plasmids expressing HEV-1 proteins were transfected into the HEK293T cells (25 ng) along with RIG-I (0.5 ng) and IFN-β firefly and TK *Renilla* luciferase reporters. RIG-I was induced with 3pdsR27 24 h post-transfection. Luciferase assay was done 16 h post-induction. **(B)** Plasmids expressing HEV-3 proteins were transfected into the HEK293T cells (25 ng) along with RIG-I (0.5 ng) and IFN-β firefly and TK *Renilla* luciferase reporters. RIG-I was induced with 3pdsR27 24 h post-transfection. Luciferase assay was done 16 h post-induction. **(C)** For IPS-1 assay, 0.5 ng of IPS-1 was co-transfected with two different quantities of HEV-1 ORF2, HEV-3 ORF2 plasmids (10 and 25 ng) or 10 ng of HEV-1 ORF3 plasmid along with IFN-β firefly and TK *Renilla* luciferase reporter plasmids. Luciferase activity was measured 24 h post-induction. **(D)** HEK293T cells were co-transfected with 0.5 ng of IPS-1 plasmid, HEV-1 ORF2 or ORF3 plasmids (25 ng each) individually or in combination along with IFN-β firefly and TK Renilla luciferase reporters. Luciferase activity was measured 24 h post-transfection. **(E)** 0.5 ng of WT RIG-I or RIG-I K858E plasmid was co-transfected with 25 ng of HEV-1 ORF2 plasmid or ORF3 plasmid along with IFN-β firefly and TK *Renilla* luciferase reporter plasmids. Induction was given 24 h post-transfection with 40 HAU/ml of Sendai virus (SeV). Luciferase activity was measured 16 h post-induction. **(F)** Effect of the HEV-1 ORF2 and ORF3 on NF-κB activity. RIG-I assay was performed by co-transfecting 0.5 ng of the RIG-I plasmid, the HEV-1 ORF2 or the HEV-1 ORF3 plasmids (25 ng) along with the NF-κB firefly and TK *Renilla* luciferase reporter plasmids. SeV infection was given 24 h post-transfection. Luciferase activity was measured 16 h post-induction. All experiments were performed in the HEK293T cells. Values are mean ± SD, *n* = 3 (^∗∗∗^ denotes *p*-values ≤ 0.001, ^∗∗^ denotes *p*-values ≤ 0.01, and ^∗^ denotes *p*-values ≤ 0.05).

In order to identify the HEV protein(s) that might be interfering with the RIG-I signaling, a reporter assay was designed in which RIG-I mediated activation of the IFN-β promoter was quantified by measuring the activity of firefly luciferase, as described earlier (Madhvi et al., 2017). For this, all the known protein domains of HEV Genotype I (HEV-1) and Genotype III (HEV-3) were cloned into the mammalian expression vector pUNO-mcs. Expression of the HEV protein domains was verified by western blotting, which confirmed their authenticity (Supplementary Figure S1). Effect of HEV-1 and HEV-3 HEV domains on RIG-I signaling was determined by measuring IFN-β promoter activation using luciferase assays. Briefly, plasmids expressing RIG-I and HEV protein domains were transfected in HEK293T cells along with reporter plasmids; firefly luciferase under IFN-β promoter and *Renilla* luciferase under thymidine kinase promoter. A synthetic 27 bp triphosphorylated double-stranded RNA agonist of RIG-I (3pdsR27) was used to induce RIG-I signaling (Ranjith-Kumar et al., 2009). Both HEV-1 and HEV-3 ORF2 inhibited RIG-I signaling (Figures 1A,B). HEV-3 PCP also inhibited RIG-I signaling as reported earlier (Nan et al., 2014b) but HEV-1 PCP did not show any inhibition (Figures 1A,B). Contrary to the earlier report, MeT domains of both genotypes showed no significant effect on RIG-I signaling (Bagdassarian et al., 2018; Kang et al., 2018). ORF3 proteins of both genotypes showed increase in IFN-β activation as reported previously (Nan et al., 2014a). We focused on characterization of the effect of HEV ORF2 on RIG-I signaling. The ORF2 protein from both genotypes showed a dose dependent inhibition of IFN-β promoter activity and HEV-1 ORF3, used as a control, showed increased activation of IFN-β promoter (Supplementary Figure S2).

To determine whether ORF2 acts directly on RIG-I, we tested its effect on IPS-1, which is the adapter protein of RIG-I. Over-expression of IPS-1 activates RIG-I signaling in the IFN-β promoter reporter assay (Kawai et al., 2005). Both HEV-1 and HEV-3 ORF2 protein lowered the IPS-1 induced IFN-β promoter activation in a dose-dependent manner whereas HEV-1 ORF3 increased the IPS-1 induced IFN-β promoter activation (Figure 1C). This result suggests that ORF2 may be inhibiting RIG-I signaling by acting at a step downstream of RIG-I. Additionally, we also looked at the effect of ORF2 on ORF3 induced IFN-β promoter activation (Figure 1D). When co-expressed in HEK293T cells, ORF2 significantly inhibited IPS-1 induced IFN-β promoter activation in the presence of ORF3, suggesting that the inhibitory effect of ORF2 is dominant.

Next, to test whether ORF2 protein could inhibit interferon response during viral infection, we infected HEK293T cells with SeV, a known inducer of RIG-I pathway (Kato et al., 2006). To determine the specificity of SeV dependent RIG-I activation, an RNA binding incompetent mutant of RIG-I, RIG-I K858E was also used (Cui et al., 2008). Expression of RIG-I and K858E mutant was confirmed by western (Supplementary Figure S1). As expected, significant activation of IFN-β promoter was observed only in case of WT RIG-I upon SeV infection (Figure 1E and Supplementary Figure S3). ORF2 inhibited SeV induced activation of the IFN-β promoter while ORF3 was unable to do so (Figure 1E). As RIG-I signaling also results in NF-κB activation, we also checked the effect of ORF2 on NF-κB promoter activity. Similar to IFN-β promoter activity, both HEV-1 and HEV-3 ORF2 protein lowered NF-κB promoter activity, further confirming its antagonistic effect on the RIG-I signaling (Figure 1F and Supplementary Figure S4). Co-expression of ORF3 in SeV infected cells resulted in 2.5-fold activation of NF-κB promoter (Figure 1F and Supplementary Figure S4). As the ORF2 proteins from both genotypes showed similar inhibition of RIG-I signaling, we performed further experiments with HEV-1 ORF2.

**FIGURE 2:**
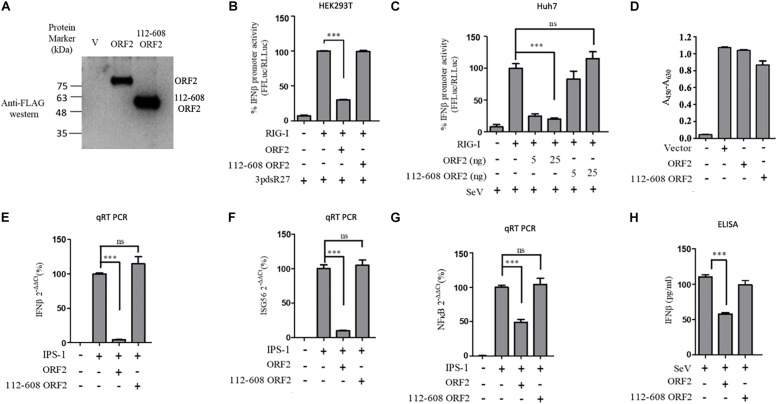
HEV 112-608 ORF2 protein does not inhibit the IFN-β promoter activation. **(A)** Western blot analysis of the HEV-1 FL ORF2 and 112-608 ORF2 in HEK293T cells. **(B)** 0.5 ng of WT RIG-I plasmid was co-transfected with 25 ng of FL ORF2 or 112-608 ORF2 plasmids along with IFN-β firefly and TK *Renilla* luciferase reporter plasmids in HEK293T cells. Induction was given 24 h post-transfection with 3pdsR27. Luciferase activity was measured 16 h post-induction. **(C)** Plasmids expressing the FL ORF2 and 112-608 ORF2 were transfected into Huh7 cells (5, 25 ng) along with RIG-I (0.5 ng) and IFN-β firefly and CMV *Renilla* luciferase reporter plasmids. Induction was given 24 h post-transfection with 100 HAU/ml of SeV. Luciferase activity was measured 16 h post-induction. **(D)** For cell viability assay, 25 ng each of vector, FL ORF2 or 112-608 ORF2 plasmids were transfected into HEK293T cells. 48 h post-transfection, WST-1 reagent (diluted 1:10 with media) was added to the cells, incubated for 1 h and absorbance (A_450_) was recorded. Values were normalized to that of the control absorbance taken at A_630_ and plotted as difference in the values (A_450_–A_630_). **(E)** HEK293T cells were co-transfected with IPS-1 (20 ng) and FL ORF2 or 112-608 ORF2 plasmids (1 μg each). RNA was isolated 24 h post transfection. Ct values corresponding to IFN-β or **(F)** ISG56 or **(G)** NF-κB mRNA levels were normalized to that of GAPDH Ct values and change in mRNA levels was calculated by the ΔΔCt method with respect to the un-induced vector control. **(H)** THP-1 cells were transfected with vector, FL ORF2, or 112-608 ORF2 plasmids. SeV infection was given 12 h post-transfection. Secreted IFN-β protein levels were estimated by a sandwich ELISA from media collected 24 h post-infection. Values are percent mean ± SD for qRT PCR and mean ± SD for ELISA. *n* = 3 for all experiments (^∗∗∗^ denotes *p*-values ≤ 0.001, ns denotes *p*-values ≥ 0.05).

Next, we tested a truncated form of the ORF2 protein, spanning the 112-608 amino acid region of the full length ORF2 protein. We first compared the expression of the full length (FL ORF2) and the truncated (112-608 ORF2) ORF2 proteins in the HEK293T cells (Figure 2A). The effect of 112-608 ORF2 protein on RIG-I signaling was assessed by IFN-β promoter reporter assays using 3PdsR27 and SeV as RIG-I inducers. In both cases, FL ORF2 inhibited IFN-β promoter activity but 112-608 ORF2 did not (Figures 2B,C). To test the observed inhibition is not due to toxic effects of the FL ORF2, cytotoxicity of these two proteins was tested in HEK293T cells, using the formazan-based reagent WST-1. Both FL ORF2 and 112-608 ORF2 did not show any significant cytotoxicity (Figure 2D). Next, transcript levels of IFN-β and a downstream interferon stimulated gene (ISG), ISG56 and NF-κB were measured by quantitative real-time PCR in the presence of FL ORF2 and 112-608 ORF2 in IPS-1 over-expressing HEK293T cells. As expected, IFN-β, ISG56, and NF-κB mRNA levels were significantly lowered in the presence of FL ORF2 but not 112-608 ORF2 (Figures 2E–G). Similar decrease in IFN-β mRNA level was observed in Huh 7 cells as well (Supplementary Figure S5). To confirm that the observed effect is reflected at the protein level as well, a sandwich ELISA was performed to measure the IFN-β protein. For this, THP1 cells transfected with FL ORF2, 112-608 ORF2 or a vector control were infected with SeV and the level of secreted IFN-β protein was measured using sandwich ELISA. IFN-β levels were calculated from a standard curve (Supplementary Figure S6A). In agreement with the result from other assays, IFN-β protein was significantly lower in the presence of FL ORF2 but not 112-608 ORF2 (Figure 2H). To ensure the expression of ORF2 and 112-608 ORF2 in THP-1 cells, a direct qualitative ELISA was performed. Purified ORF2 protein was used as positive control. Ectopically expressed ORF2 and 112-608 ORF2 proteins were expressed at similar levels (Supplementary Figure S6B).

### HEV-1 ORF2 Glycosylation or Dimerization Has No Effect on Its Ability to Antagonize the RIG-I Signaling

**FIGURE 3:**
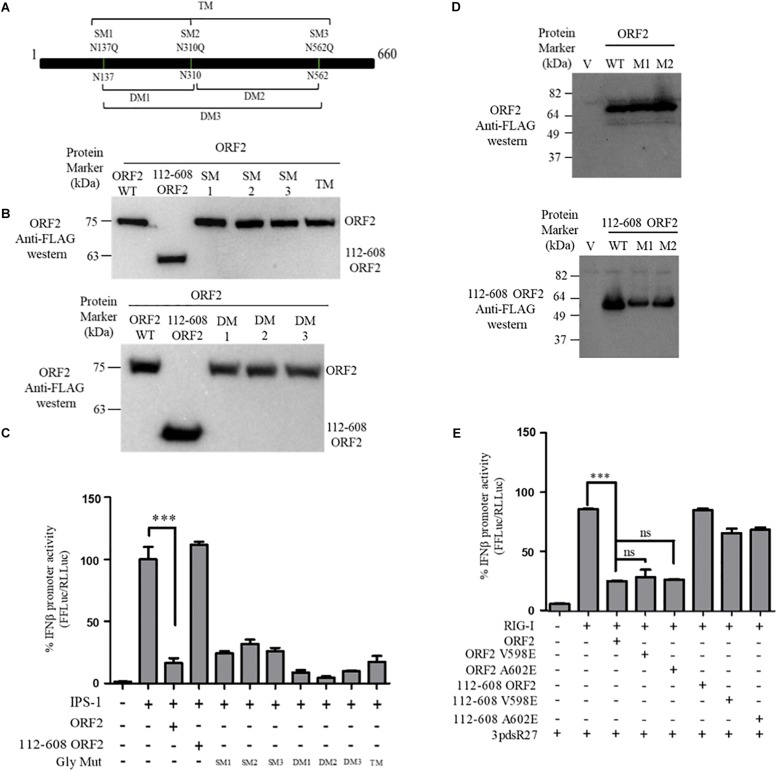
ORF2 glycosylation and dimerization is not required for IFN-β inhibition: **(A)** A schematic showing the location of the three glycosylation sites and the mutations introduced in the FL ORF2. **(B)** Western blot analysis of WT and mutant FL ORF2 or 112-608 ORF2 in HEK293T cells. **(C)** For IPS-1 assay, 0.5 ng of IPS-1 was co-transfected with 25 ng of WT or mutant FL ORF2 or 112-608 ORF2 plasmids along with IFN-β firefly and TK *Renilla* luciferase reporter plasmids. Luciferase activity was measured 24 h post-induction. **(D)** Western blot analysis of the whole cell lysates of HEK293T cells expressing the WT FL ORF2 (top) or 112-608 ORF2 (bottom) and their two mutants, V598E (M1) and A602E (M2) ORF2. **(E)** RIG-I assay was performed with RIG-I (0.5 ng) and 25 ng of the WT and mutant ORF2 plasmids. Induction was given by transfecting 3pdsR27 24 h post-transfection. Luciferase activity was measured 16 h post-induction. Values are percent mean ± SD, *n* = 3 (^∗∗∗^ denotes *p*-values ≤ 0.001, ns denotes *p*-values ≥ 0.05).

FL ORF2 is glycosylated and 112-608 ORF2 is unglycosylated (Zafrullah et al., 1999; Jimenez de Oya et al., 2012). To test the role of glycosylation in the observed inhibition of IFN-β production by the FL ORF2 protein, we created glycosylation deficient mutants of FL ORF2. All three reported glycosylation sites of FL ORF2, such as N137, N310, and N562 (Zafrullah et al., 1999) were mutated individually (single mutants, SM), in pairs (double mutants, DM), or simultaneously (triple mutant, TM) (Figure 3A). Western blot analysis revealed that expression of all the mutants was quite similar to that of the wild type ORF2 (Figure 3B). In the IFN-β promoter reporter assay, none of the glycosylation mutants showed any significant difference compared to the wild type ORF2 in inhibiting the IPS-1 induced RIG-I signaling (Figure 3C).

HEV ORF2 protein self-associates and forms higher order structures (Li et al., 2009). To understand whether dimerization played any role in the inhibition of IFN-β promoter activation by FL ORF2, dimerization deficient mutants V598E and A602E were generated in FL ORF2 and 112-608 ORF2, based on a previously reported study (Li et al., 2009). The mutations did not affect the expression levels of these proteins (Figure 3D). Next, the effect of these mutants on RIG-I signaling was analyzed. IFN-β promoter reporter assay revealed that the V598E and A602E mutations inhibited 3pdsR27 induced RIG-I signaling, similar to the wild type ORF2 (Figure 3E). These data suggest that neither glycosylation nor higher order structures of FL ORF2 are a prerequisite for its observed inhibitory activity.

### HEV-1 ORF2 Inhibits TRIF and MyD88 Induced IFN-β Production

**FIGURE 4:**
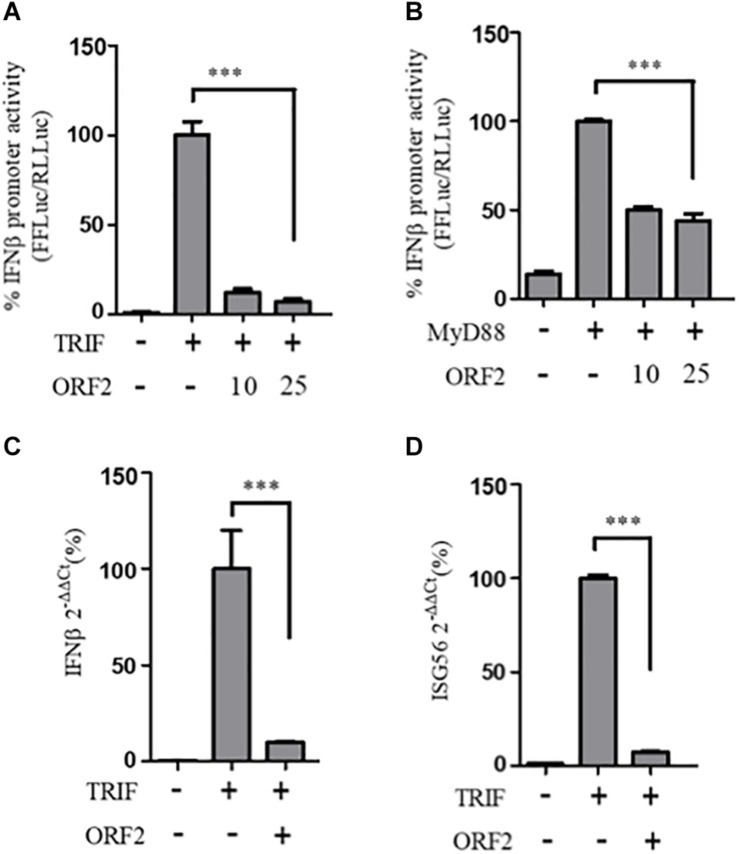
HEV ORF2 protein inhibits TRIF and MyD88 induced IFN-β promoter activation. **(A)** TRIF assay was performed by co-transfecting 0.5 ng myc-TRIF plasmid with two different quantities of FL ORF2 (10 and 25 ng) along with IFN-β firefly and TK *Renilla* luciferase reporter plasmids. Luciferase activity was measured 24 h post-induction. **(B)** MyD88 assay was performed by co-transfecting 2.5 ng of MyD88 plasmid with two different quantities of FL ORF2 (10 and 25 ng) along with IFN-β firefly and TK *Renilla* luciferase reporter plasmids. Luciferase activity was measured 24 h post-induction. **(C)** Cells were co-transfected with TRIF (20 ng) and FL ORF2 plasmids (1 μg each). RNA was isolated 24 h post transfection. Ct values corresponding to IFN-β and **(D)** The ISG56 mRNA levels were normalized to that of the GAPDH Ct values and change in the mRNA level was calculated by the ΔΔCt method with respect to the un-induced vector control. All experiments were performed in HEK293T cells. Values are percent mean ± SD, *n* = 3 for all experiments (^∗∗∗^ denotes *p*-values ≤ 0.001).

To determine whether the observed inhibition of IFN-β promoter activity is specific to the RIG-I signaling pathway, IFN-β production through Toll-like receptor (TLR) adapters TRIF and MyD88 were analyzed in the presence and absence of HEV-1 ORF2 (Figure 4). Note that TRIF is an adapter for TLR3 and all other TLRs use MyD88. TLR4 can signal via both TRIF and MyD88 (Lester and Li, 2014). First, the expression of these adapter proteins was confirmed by western (Supplementary Figure S1). FL ORF2 inhibited TRIF and MyD88 induced IFN-β promoter activity (Figures 4A,B). TRIF induced IFN-β and ISG56 mRNA levels were also significantly reduced in the presence of FL ORF2 (Figures 4C,D) suggesting that ORF2 interferes with TLR signaling pathways.

### IRF3 Nuclear Translocation Is Affected in the Presence of HEV FL ORF2

**FIGURE 5:**
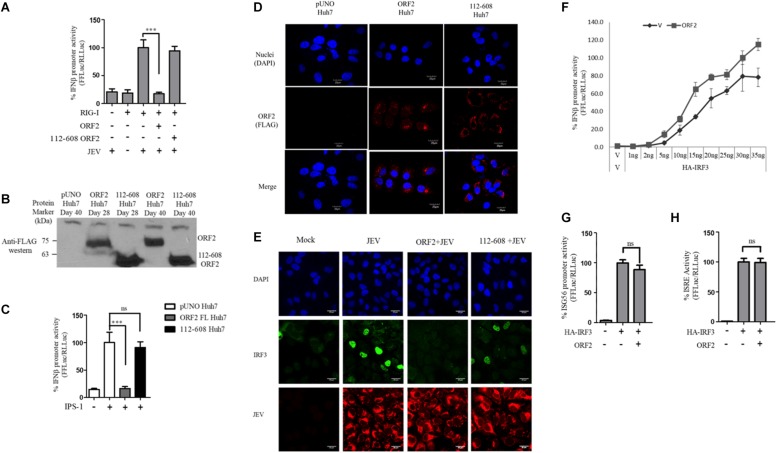
IRF3 nuclear translocation is inhibited by the HEV ORF2 protein: **(A)** RIG-I assay was performed by co-transfecting RIG-I (0.5 ng) and FL ORF2 or 112-608 ORF2 plasmids with IFN-β firefly and CMV *Renilla* luciferase reporter plasmids in Huh7 cells. JEV infection was given at the 0.5 MOI for 4 h. Luciferase activity was measured 16 h post-infection. **(B)** Stable expression of FL ORF2 and 112-608 ORF2 proteins was monitored by western blot analysis. Cell lysates at day 28 and day 40 for each of ORF2 Huh7 and 112-608 Huh7 were probed with anti-FLAG antibody. **(C)** For IPS-1 assay, IPS-1 (5 ng) and IFN-β firefly and CMV *Renilla* luciferase reporter plasmids were transfected in pUNO Huh7, ORF2 Huh7, and 112-608 Huh7 stable cell lines. IFN-β promoter activity was measured 24 h post-transfection. **(D)** pUNO Huh7, ORF2 Huh7 and 112-608 Huh7 cells were treated with the goat anti-FLAG primary antibody and anti-Goat Alexa-568 (red) secondary antibody to visualize the ORF2 proteins. Nuclei were stained with DAPI. The scale represents 20 μm. **(E)** pUNO Huh7, ORF2 Huh7, and 112-608 Huh7 cells were infected with the JEV at 0.5 MOI for 24 h. Nuclei were stained with DAPI, IRF3 was stained with rabbit anti-IRF3 primary and anti-rabbit Alexa-488 (green) antibody and JEV was stained with mouse anti-JEV glycoprotein E antibody and anti-mouse Alexa-596 (red) antibody. **(F)** IRF3 assay was performed by co-transfecting increasing quantities of HA-IRF3 and 25 ng of FL ORF2 plasmids with IFN-β firefly and TK *Renilla* luciferase reporter plasmids. Luciferase activity was measured 24 h post-transfection. **(G)** IRF3 assay was performed by co-transfecting HA-IRF3 and 25 ng of FL ORF2 plasmids with ISG56 firefly and TK *Renilla* luciferase reporter plasmids. Luciferase activity was measured 24 h post-transfection. **(H)** IRF3 assay was performed by co-transfecting HA-IRF3 and 25 ng of FL ORF2 plasmids with ISRE firefly and TK *Renilla* luciferase reporter plasmids. Luciferase activity was measured 24 h post-transfection. Values are mean ± SD, *n* = 3 for all experiments. (^∗∗∗^ denotes *p*-values ≤ 0.001, ns denotes *p*-values ≥ 0.05).

One of the proteins central to both the RIG-I and the TLR pathway is the transcription factor IRF3, which when activated undergoes phosphorylation, dimerization, and subsequent nuclear translocation (Seth et al., 2006). In order to monitor IRF3 nuclear translocation in the presence and absence of the FL and 112-608 ORF2 proteins, an immunofluorescence assay was performed. Due to the lack of SeV specific antibody for detection, JEV, which is also a known agonist of RIG-I pathway, was used as an inducer (Chang et al., 2006). We first verified RIG-I pathway activation by JEV by measuring the RIG-I induced IFN-β promoter reporter activity. Infection with JEV led to a robust induction of IFN-β promoter activity, only when RIG-I was co-expressed, indicating that the RIG-I pathway is activated upon JEV infection. As with SeV, JEV induced IFN-β promoter activation was inhibited by the FL ORF2 but not the 112-608 ORF2 (Figure 5A).

Transient transfection often results in differential levels of expression in different cells depending on the transfection efficiency. Therefore, we established a hepatoma stable cell line that would constitutively express the FL ORF2 (ORF2 Huh7) or 112-608 ORF2 (112-608 Huh7) proteins. A vector control (pUNO Huh7) cell line was also generated. These cell lines were validated for the expression of the ORF2 proteins by western blotting (Figure 5B). The cell lines showed robust expression of FL ORF2 and 112-608 ORF2 proteins, which was consistently seen for more than 2 months (Figure 5B).

Next, the ability of stably expressed FL ORF2 to inhibit interferon response was determined by measuring the level of IPS-1 induced IFN-β promoter activation. For this, ORF2 Huh7, 112-608 Huh7, and pUNO Huh7 cell lines were transfected with IPS-1 and IFN-β promoter reporter plasmids. IFN-β promoter driven luciferase activity was significantly reduced in ORF2 Huh7 cells, but not in 112-608 Huh7 cells, keeping with the results obtained with transient transfection assays (Figure 5C). An immunofluorescence experiment was performed to check whether FL ORF2 and 112-608 ORF2 were expressed in all the cells at a comparable level. The ORF2 proteins were visualized with an anti-FLAG antibody and an Alexa-568 conjugated secondary antibody. Most of the cells showed similar levels of the two ORF2 proteins (Figure 5D middle column and right column). Next, these cells were infected with JEV to induce IRF3 phosphorylation and nuclear localization. A mock infection control was also used where pUNO Huh7 cells were incubated only with the infection media without JEV (Figure 5E, Mock).

High levels of IRF3 nuclear localization could be seen in control cells infected with JEV (Figure 5E, JEV) compared to mock. JEV infection was detected using antibody specific to its Glycoprotein E. However, in ORF2 Huh7 cells, IRF3 nuclear localization was significantly reduced (Figure 5E, ORF2 + JEV). Significant IRF3 translocation was observed in 112-608 Huh7 cells (Figure 5E, 112-608 + JEV). These data indicate that FL ORF2 inhibits translocation of IRF3 into nucleus.

To check whether ORF2 is directly acting on IRF3, we tested the effect of FL ORF2 on HEK293T cells overexpressing IRF3. FL ORF2 was unable to inhibit IRF3 overexpression-induced activation of the IFN-β promoter (Figure 5F). Similar result was obtained in IRF3 overexpression-induced activation of the ISG56 and ISRE promoters (Figures 5G,H, respectively). Taken together, these data suggest that the FL ORF2 may be acting at a site down stream of adaptor proteins of RLR and TLR pathways but upstream of IRF3.

## Discussion

Rapid recognition of pathogens by pathogen recognition receptors is essential for mounting an effective immune response against it. Viruses have evolved multiple strategies to counter the innate immune recognition to replicate and spread efficiently. RIG-I is one of the key PRRs that recognize HEV (Xu et al., 2017). Earlier reports have shown that X, PCP, and MeT domains of HEV interfere with RIG-I signaling (Nan et al., 2014b; Bagdassarian et al., 2018; Kang et al., 2018). To identify whether other HEV encoded proteins inhibit interferon response, we ectopically expressed each protein domains of ORF1 along with ORF2 and ORF3 and assessed their ability to modulate RIG-I signaling. In our initial screen of HEV proteins, we identified ORF2 as a RIG-I inhibitor protein along with HEV-3 PCP. Compared to PCP, inhibition by ORF2 was higher and was seen in both genotypes, HEV-1 and HEV-3. These results point to an auxiliary role of the ORF2 protein in addition to encapsidation as a host immune regulator protein.

The full length ORF2 protein was previously shown to undergo processing to form the 112-608 aa product when expressed in insect cell lines (Zhang et al., 1997). It was hypothesized that the processed protein forms the viral capsid and is structurally similar to the full length ORF2 protein (Li et al., 1997). However, the 112-608 ORF2 did not inhibit IFN-β or NF-κB promoter activity whereas inhibition was consistently observed with the FL ORF2 protein. Interference of the RIG-I signaling was confirmed in different cell lines, using synthetic and viral RIG-I agonists, by reporter assays involving different interferon inducible promoters, qRT PCR, and ELISA and immunofluorescence microscopy. This difference between the effect of FL ORF2 and 112-608 ORF2 proteins on the RIG-I signaling could be due to lack of the N-terminal 111 or the C-terminal 52 amino acids in the 112-608 ORF2 protein. Studies have shown that the 112-608 aa region of the ORF2 forms T = 1 particles that are structurally distinct from the T = 3 native particles and do not encapsidate the viral RNA (Yamashita et al., 2009). Thus, the apparent lack of inhibition by 112-608 ORF2 could also be due to change in the structure.

Absence of the first 111 amino acids in 112-608 ORF2 also suggests that the protein lacks the signal sequence that translocates the protein to endoplasmic reticulum and hence is not glycosylated. Three potential glycosylation sites were identified for the HEV ORF2 protein (Zafrullah et al., 1999). Mutating these sites hindered capsid assembly and virus infectivity (Graff et al., 2008). Mutating the glycosylation sites did not alter the FL ORF2 protein induced IFN-β inhibition. This could mean that the structural features required for the FL ORF2 induced IFN-β inhibition are not altered in the absence of glycosylation.

In order to protect their genome, viral capsid proteins must assemble in a tightly packed capsid. Hence, the capsid proteins display strong self-interactions and often form dimers and hexamers, before the final assembly into mature virions, with or without the help of host proteins. It was shown that dimerization of the ORF2 protein plays a crucial role in recognition of the protein by host antibodies (Li et al., 2009). Thus, two of the residues (V598 and A602) that were shown to be critical for dimerization were mutated in FL ORF2 and 112-608 ORF2. V598 and A602 in context of 112-608 ORF2 were able to inhibit IFN-β promoter activity better than 112-608 ORF2. This could be due to altered structure of these mutants compared to that of 112-608 ORF2. However, this difference was not significantly high and was entirely absent in ORF2 indicating that formation of higher order structures by ORF2 may not be required for RIG-I antagonism.

While our manuscript was being prepared, an article was published, which showed a direct interaction of HEV ORF2 with TBK-1 (Lin et al., 2019). The authors suggest that ORF2 interacts with TBK-1 via its N-terminal arginine rich region and interferes with the RIG-I/MDA5 pathway. Our data further show that in addition to RLR HEV ORF2 also affects pathway TLR pathway. Analyses with adapter proteins of the RIG-I-like receptors (RLRs) and TLR pathways, IPS-1, TRIF, and MyD88 showed that the ORF2 protein inhibited signaling via these PRRs. Multiple studies have shown that MyD88 signaling pathway does not involve TBKI (Clark et al., 2011; Kawasaki and Kawai, 2014). Inhibition of MyD88 signaling by ORF2 suggests that it may be acting multiple sites in addition to TBK1. This suggests that ORF2 may be acting at a site common to multiple innate immune receptors. There is an inherent redundancy in different immune pathways as they converge downstream of the adaptors and utilize the same effector molecules for signal transduction (Takeuchi and Akira, 2010).

Both RLR and TLR families induce type I IFN production through a family of interferon regulatory factors (IRFs) (Fischer et al., 2010). In particular, IRF3 gets phosphorylated and forms a dimer, which then translocates to the nucleus and drives type I IFN transcription by binding to the interferon stimulated regulatory elements (ISREs) situated in the promoters of all the ISGs (Fischer et al., 2010). FL ORF2 was able to lower nuclear localization of IRF3 but it could not inhibit IRF3 over-expression induced IFN-β promoter activity. Taken together, these data indicate that the ORF2 protein targets innate immune signaling downstream of adapter proteins and upstream of the IRF3. As both IFN-β and NF-κB axes of the RIG-I signaling and the TLR signaling are affected, the HEV ORF2 protein most likely brings about a universal suppression of the innate immune response. Further studies are required to understand the underlying mechanisms and identify the host proteins involved in the inhibition.

## Data Availability Statement

All datasets generated for this study are included in the article/Supplementary Material.

## Author Contributions

SH and CR-K did experimental design and data analysis. SH, NJ, and CR-K performed the experiments. MS provided material, analysis tools, and suggestions. SH, MS, and CR-K wrote the manuscript.

## Conflict of Interest

The authors declare that the research was conducted in the absence of any commercial or financial relationships that could be construed as a potential conflict of interest.
