# Supplementary material for: Hepatitis E Virus ORF2 Inhibits RIG-I Mediated Interferon Response
Source: Front Microbiol. 2020 Apr 15;11:656. doi: 10.3389/fmicb.2020.00656 (PMC7174656; doi:10.3389/fmicb.2020.00656)
Supplement: Supplementary file 1 [file Data_Sheet_1.docx]

Supplementary Material


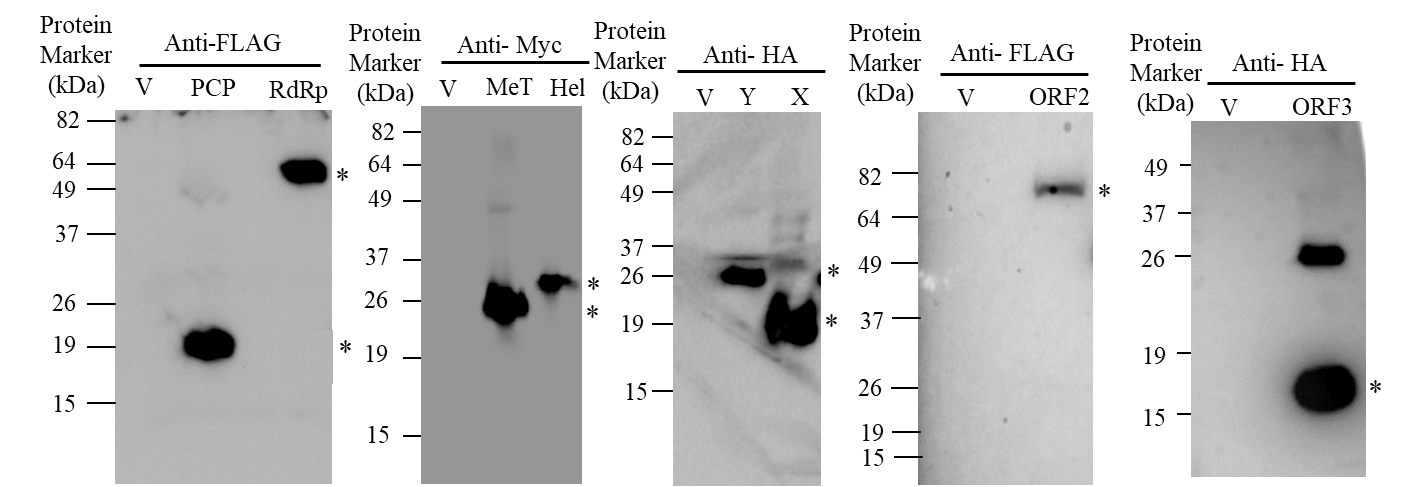


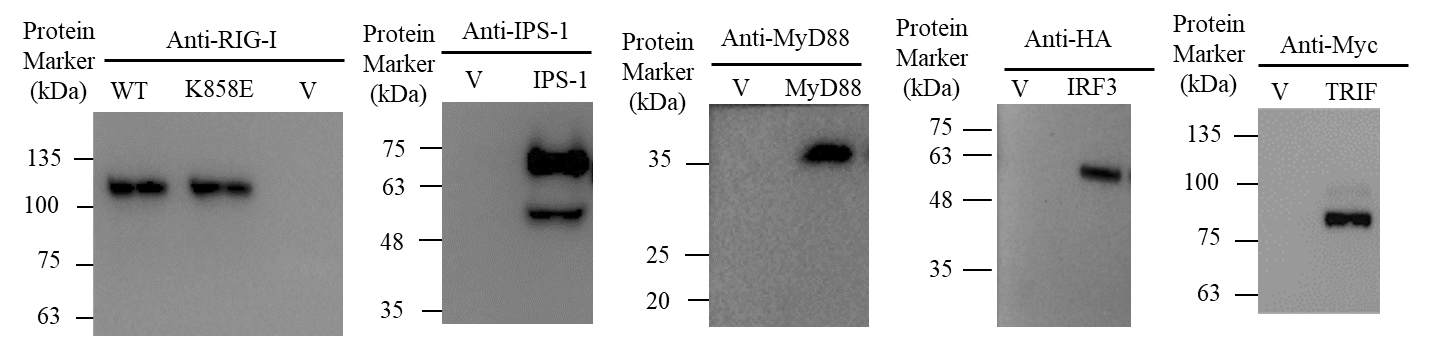


**Supplementary figure 1:** Expression of HEV-1 proteins and innate immune signaling proteins: Western blotting was performed 48 h post-transfection. For HEV, proteins were separated in individual blots as per their tags and probed with antibodies specific for that tag. (PCP, RdRp and ORF2 with anti-FLAG antibody, MeT and helicase with anti-myc antibody and Domains X, Y and ORF3 with anti-HA antibody) * denotes specific bands. For the innate immune signaling proteins, antibodies specific for the proteins were used in case of RIG-I, IPS-1 and MyD88. HA-IRF3 was visualized using anti-HA antibody and myc-TRIF was visualized using anti-Myc antibody.

**Supplementary figure 2:** Decrease in IFN-β promoter activity with HEV ORF2: Plasmids expressing HEV-1 and HEV-3 ORF2 and ORF3 were transfected in HEK293T (10 ng, 25 ng) with RIG-I (0.5 ng), IFN-β firefly and TK *Renilla* luciferase reporters. RIG-I induction was with 3pdsR27 24 h post-transfection. Activity was measured 16 h post-induction. (*** denotes p-values ≤ 0.001).

**Supplementary figure 3:** HEV-3 ORF2 inhibits Sendai Virus induced RIG-I signaling: RIG-I assay performed with RIG-I WT, K270A or K858E mutant (0.5 ng) in HEK293T cells in the presence of HEV-3 ORF2 (25 ng) and ORF3 (25 ng) and IFN-β firefly and TK *Renilla* luciferase reporters. RIG-I was induced with SeV at 40 HAU/ml 24 h post-transfection. Luciferase assay was done 16 h post-induction. (*** denotes p-value ≤ 0.001).

**
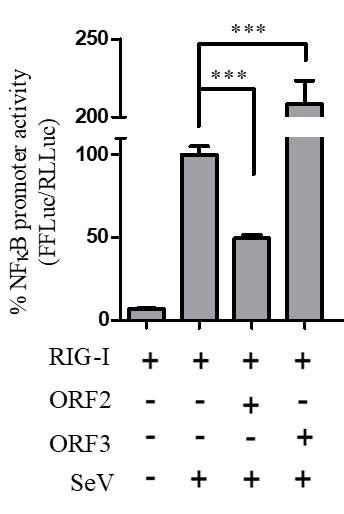
**

**Supplementary figure 4:** Effect of the HEV-3 ORF2 and ORF3 on NF-κB activity. RIG-I assay was performed by co-transfecting 0.5 ng of the RIG-I plasmid, the HEV-3 ORF2 or the HEV-3 ORF3 plasmids (25 ng) along with the NF-κB firefly and TK *Renilla* luciferase reporter plasmids. SeV infection was given 24 h post-transfection. Luciferase activity was measured 16 h post-induction. All experiments were performed in the HEK293T cells. Values are mean ± SD, n = 3. (*** denotes p-values ≤ 0.001).


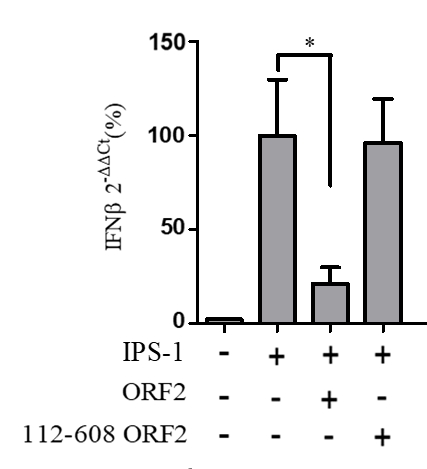


**Supplementary figure 5:** HEV-1 FL ORF2 lowers IFN-β transcription**:** Huh7 Cells were co-transfected with IPS-1 (20 ng) and HEV-1 ORF2 or 112-608 ORF2 plasmids (1 μg each). RNA was isolated 24 h post transfection. Ct values corresponding to IFN-β mRNA levels were obtained and normalized to GAPDH Ct values of the same sample and change in mRNA levels was calculated by the ΔΔCt method with respect to the un-induced vector control.

B

A


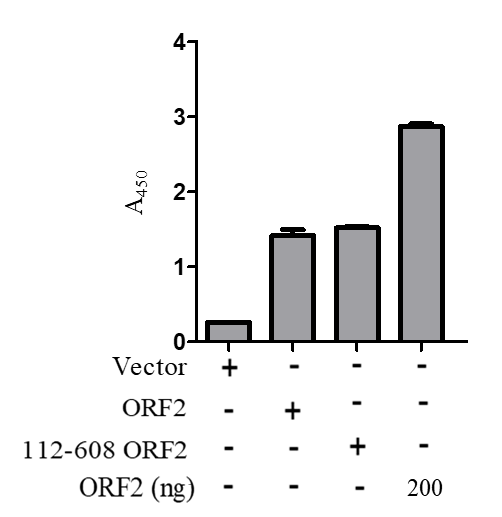


**Supplementary figure 6: (A)** Standard curve obtained for quantification of secreted IFN-β protein. Purified IFN-β protein provided in the ELISA kit was diluted in the concentrations mentioned. **(B)** ORF2 and 112-608 ORF2 expression in THP-1 cells was validated using direct qualitative ELISA. A rabbit polyclonal anti-ORF2 antibody was used to detect the ORF2 proteins. 200 ng of purified ORF2 protein was used as positive control.

**Supplementary table 1: List of plasmids**

| **Plasmids** | **Description** |
| --- | --- |
| pUNO-RIG-I | Purchased from Invivogen. |
| pUNO-RIG-I K858E | Cloned in pUNO MCS between AgeI-NheI sites. Single point mutations introduced at site K858 to E858 by site directed mutagenesis. |
| pUNO-IPS-1 | Purchased from Invivogen. |
| pUNO HEV-1 MeT myc | Genotype I HEV MeT amplified from pSK HEV-1 cDNA clone. Cloned in pUNO-MCS between AgeI- NheI sites with a C-terminal myc tag. |
| pUNO HEV-1 Y HA | Genotype I HEV Y-domain amplified from pSK HEV-1 cDNA clone. Cloned in pUNO-MCS between BamHI - PstI sites with a C-terminal HA tag. |
| pUNO HEV-1 PCP FLAG | Genotype I HEV PCP amplified from pSK HEV-1 cDNA clone. Cloned in pUNO-MCS between AgeI- NheI sites with a C-terminal FLAG tag. |
| pUNO HEV-1 X HA | Genotype I HEV X-domain amplified from pSK HEV-1 cDNA clone. Cloned in pUNO-MCS between BamHI - PstI sites with a C-terminal HA tag. |
| pUNO HEV-1 Helicase myc | Genotype I HEV Helicase amplified from pSK HEV-1 cDNA clone. Cloned in pUNO-MCS between AgeI - NheI sites with a C-terminal myc tag. |
| pUNO HEV-1 RdRp FLAG | Genotype I HEV RdRp amplified from pSK HEV-1 cDNA clone. Cloned in pUNO-MCS between AgeI - NheI sites with a C-terminal FLAG tag. |
| pUNO HEV-1 ORF3 HA | Genotype I HEV ORF3 amplified from pSK HEV-1 cDNA clone. Cloned in pUNO-MCS between AgeI - NheI sites with a C-terminal HA tag. |
| pUNO HEV-1 ORF2 FLAG | Genotype I HEV ORF2 (1-660aa) amplified from pSK HEV-1 cDNA clone. Cloned in pUNO-MCS between BamHI - PstI sites with a C-terminal FLAG tag. |
| pUNO HEV-1 ORF2 112-608 FLAG | Genotype I HEV ORF2 112-608 (112-608 aa) amplified from HEV-1 ORF2 FLAG clone. Cloned in pUNO-MCS between BamHI - PstI sites with a C-terminal FLAG tag. |
| HEV-3 MeT myc | Genotype III HEV ORF2 MeT amplified from pSK HEV-3 cDNA clone. Cloned in pUNO-MCS between AgeI - NheI sites with a C-terminal myc tag. |
| HEV-3 Y HA | Genotype III HEV Y-domain amplified from pSK HEV-3 cDNA clone. Cloned in pUNO-MCS between AgeI - NheI sites with a C-terminal HA tag. |
| HEV-3 PCP FLAG | Genotype III HEV PCP amplified from pSK HEV-3 cDNA clone. Cloned in pUNO-MCS between AgeI - NheI sites with a C-terminal FLAG tag. |
| HEV-3 X HA | Genotype III HEV X-domain amplified from pSK HEV-3 cDNA clone. Cloned in pUNO-MCS between AgeI - NheI sites with a C-terminal FLAG tag. |
| HEV-3 Helicase myc | Genotype III HEV Helicase amplified from pSK HEV-3 cDNA clone. Cloned in pUNO-MCS between AgeI - NheI sites with a C-terminal myc tag. |
| HEV-3 RdRp FLAG | Genotype III HEV RdRp amplified from pSK HEV-3 cDNA clone. Cloned in pUNO-MCS between AgeI - NheI sites with a C-terminal FLAG tag. |
| HEV-3 ORF3 HA | Genotype III HEV ORF3 amplified from pSK HEV-3 cDNA clone. Cloned in pUNO-MCS between AgeI - NheI sites with a C-terminal HA tag. |
| HEV-3 ORF2 FLAG | Genotype III HEV ORF2 (1-660aa) amplified from pSK HEV-3 cDNA clone. Cloned in pUNO-MCS between AgeI - NheI sites with a C-terminal FLAG tag. |
| IFNβ firefly Luc reporter plasmid | Kind gift from Dr. R. Lin, McGill University, Canada. |
| phRLTKLuc *renilla* reporter plasmid | Purchased from Promega |
| cmvRLLuc *renilla* reporter plasmid | Purchased from Promega |
| pUNO-IRF3 HA | Amplified from a commercially available vector with an HA-tag at C-terminus. ` |
| pCDNA-TRIF myc | Kind gift from Dr. Stanley Lemon, University of North Carolina, USA |
| pUNO MyD88 | Purchased from Invivogen |
| pUNO-C1QBP myc | Amplified from Huh7 cDNA with a myc-tag at C-terminus. Cloned in pUNO-MCS between AgeI - NheI sites with a C-terminal FLAG tag. |

**Supplementary table 2: List of primers**

| **Name** | **Sequence** |
| --- | --- |
| HEV-1 MeT F | GACACCGGTCATCATGGAGGCCCATCAGTTTATCAAGGC |
| HEV-1 MeT myc R | GACGCTAGCTCACAGGTCTTCTTCAGAGATCAGTTTCTGTTCAATCCAGGAGCGCAGGTTGGAAACATCG |
| HEV-1 Y F | GACACCGGTCATCATGCGCGTTGTGGTGACGTATGAGG |
| HEV-1 Y HA R | GACGCTAGCTCAAGCGTAATCTGGAACATCGTATGGGTAAAAGCCGGCCGAGAGCCAGCGCCTACAC |
| HEV-1 PCP F | GACACCGGTCATCATGCAGTGTAGGCGCTGGCTCTC |
| HEV-1 PCP FLAG R | GACGCTAGCTCACTTATCGTCATCGTCCTTGTAGTCGAGATTGTGGCGCTCTGG |
| HEV-1 X F | GACACCGGTCATCATGCCGGATGGCTCTAAGGTGTTCGCC |
| HEV-1 X HA R | GACGCTAGCTCAAGCGTAATCTGGAACATCGTATGGGTATGCTGTCCGCGCAACATCCTCAGTTATAG |
| HEV-1 hel F | GACACCGGTCATCATGGGCTGTCGAGTCACCCCCG |
| HEV-1 hel myc R | GACGCTAGCTCACAGGTCTTCTTCAGAGATCAGTTTCTGTTCGAAAAAGTTATTAACGATTGCATCGGAG |
| HEV-1 RdRp F | GACACCGGTCATCATGGGTGGCGAAATTGGCCACC |
| HEV-1 RdRp FLAG R | GACGCTAGCTCACTTATCGTCATCGTCCTTGTAGTCTTCCACCCGACACAGAATTG |
| HEV-1 ORF3 F | GACACCGGTCATCATGGGTTCGCGACCATGCG |
| HEV-1 ORF3 R HA | GACGCTAGCTTAAGCGTAATCTGGAACATCGTATGGGTAGCGGCGCGGCCCCAGCTG |
| HEV-1 ORF2 BamHI F | GACGGATCCCATCATGCGCCCTCGGCCTATTTTGC |
| HEV-1 Flag ORF2 660R PstI | GACCTGCAGCTACTTATCGTCATCGTCCTTGTAGTCTAACTCCCGAGTTTTACCCACC |
| HEV-3 MeT BamHI F | GACGGATTCCATCATGGAGGCCCACCAGTTCATTAAGGC |
| HEV-3 MeT myc R | GACGCTAGCTCACAGGTCTTCTTCAGAGATCAGTTTCTGTTCGATCCACGCACGAAGTATGGAAACATCATGG |
| HEV-3 Y F | GACACCGGTCATCATGCGCGCCGTTGTAACCTATGAGGGCG |
| HEV-3 Y HA R | GACGCTAGCTCAAGCGTAATCTGGAACATCGTATGGGTAGAATCCTGCAGATAGCCACCGTCGG |
| HEV-3 protease F | GACACCGGTCATCATGCAGTGCCGACGGTGGCTATC |
| HEV-3 protease FLAG R | GACGCTAGCTCACTTATCGTCATCGTCCTTGTAGTCCAGGACATACTGCTCTGG |
| HEV-3 X F | GACACCGGTCATCATGCCCGACGGCGCTAAGGTGTATGCAG |
| HEV-3 X HA R | GACGCTAGCTCAAGCGTAATCTGGAACATCGTATGGGTAGGCCGTACGAGCCGTATCCTCAGTTATGG |
| HEV-3 hel F | GACACCGGTCATCATGGGCTGCACTATCAGTCCTGG |
| HEV-3 hel myc R | GACGCTAGCTCACAGGTCTTCTTCAGAGATCAGTTTCTGTTCGAAAAAATTATTGACAATCACATCCGAG |
| HEV-3 RdRp F | GACACCGGTCATCATGGGCGGAGAGGTCGGCCATCACCGCCCTTC |
| HEV-3 RdRp Flag R | GACGCTAGCTCACTTATCGTCATCGTCCTTGTAGTCTTCTACCCGCTGTATGATGGAATTTG |
| HEV-3 ORF3 F | GACACCGGTCATCATGGGATCACCATGTGCCC |
| HEV-3 ORF3 R HA | GACGCTAGCTCAAGCGTAATCTGGAACATCGTATGGGTAACGGCGCAGCCCCAGCTGG |
| HEV-3 ORF2 F | GACACCGGTCATCATGTGCCCTAGGGTTGTTCTGC |
| HEV-3 ORF2 Flag 660R NheI | GACGCTAGCTTACTTATCGTCATCGTCCTTGTAGTCAGACTCCCGGGTTTTGCCTACC |
| HEV-1 ORF2 112F BamHI | GACGGATCCCATCATGGCGGTCGCTCCGGCCCATGAC |
| HEV-1 Flag ORF2 608R PstI | GACCTGCAGCTACTTATCGTCATCGTCCTTGTAGTCTGCTAGCACAGAGTGGGGGGCTAAAAC |

**Supplementary table 3:** List of mutagenesis primers

| **Mutagenesis Primers** | **Sequence** |
| --- | --- |
| HEV-1 ORF2 N137Q F | CGC CGG CAG TAT CAA CTA TCA ACA TCT CC |
| HEV-1 ORF2 N137Q R | GGA GAT GTT GAT AGT TG A TAC TGC CGG CG |
| HEV-1 ORF2 N310Q F | GAA CTT GAG TTC CGC CAA CTC ACC CCC GGT AAT AC |
| HEV-1 ORF2 N310Q R | GT ATT ACC GGG GGT GAG TTG GCG GAA CTC AAG TTC |
| HEV-1 ORF2 N562Q F | CCCT TAT AAT TAT CAA ACC ACT GCT AGT GAC C |
| HEV-1 ORF2 N562Q R | GGT CAC TAG CAG TGG TTT GAT AAT TAT AAG GG |
| HEV-1 ORF2 V598E F | C GTC TCT ATT TCC GCG GAG GCT GTT TTA GCC C |
| HEV-1 ORF2 V598E R | G GGC TAA AAC AGC CTC CGC GGA AAT AGA GAC G |
| HEV-1 ORF2 A602E F | GCG GTT GCT GTT TTA GAG CCC CAC TCT GTG C |
| HEV-1 ORF2 A602E R | G CAC AGA GTG GGG CTC TAA AAC AGC AAC CGC |

**Supplementary table 4:** List of qRT primers

| **Name** | **Sequence** |
| --- | --- |
| IFNb L F | ACGCCGCATTGACCATCTAT |
| IFNb L R | TTGGCCTTCAGGTAATGCAGA |
| GAPDH L F | GGTGAAGGTCGGAGTCAACG |
| GAPDH L R | AGGGATCTCGCTCCTGGAAG |
| ISG56 L F | GGCCTTGCTGAAGTGTGGAG |
| ISG56 L R | GCTTCAGGGCAAGGAGAACC |
| NFkB L F | GGTCAAGGAGCTGCAGGAGA |
| NFkB L R | ACAGCCAGCTCCCAGAAGTG |
